# Surface Roughening Behavior and Mechanism in Aluminum Alloy Under Tensile Deformation

**DOI:** 10.3390/ma17235911

**Published:** 2024-12-03

**Authors:** Xiang Zeng, Shaoming Xu, Zhongbao Mi, Leheng Huang, Xuefeng Xu, Yubin Fan, Jiawen Yu, Xiaoguang Fan, Xiaoxiao Chen, Qiqi Tu

**Affiliations:** 1 School of Aeronautical Manufacturing and Mechanical Engineering, Nanchang Hangkong University, Nanchang 330063, China; yanyeqikong@163.com (S.X.); mi09050427@163.com (Z.M.); h13907072473@126.com (L.H.); xxf@nchu.edu.cn (X.X.); yubin_fan@nchu.edu.cn (Y.F.); 2 Jiangxi Key Laboratory of Extreme Manufacturing Technology for High-End Equipment, Nanchang Hangkong University, Nanchang 330063, China; 3 Shaanxi Key Laboratory of High-Performance Precision Forming Technology and Equipment, Northwestern Polytechnical University, Xi’an 710072, China; garvenyu@mail.nwpu.edu.cn; 4 Guizhou Aerospace Precision Products Co., Ltd., Zunyi 563006, China; 15985248085@sina.cn (X.C.); tq17830677471@126.com (Q.T.)

**Keywords:** 2219 aluminum alloy, surface roughening, tensile deformation, solid solution treatment, crystal plasticity simulation

## Abstract

Surface roughening (SR) has been found to occur in solid solution 2219 aluminum alloy under tensile deformation, which will deteriorate its surface quality. To make a precise study of the surface roughening (SR) behavior and mechanism, the surface morphology of annealed and solid solution 2219 aluminum alloy was compared and crystal plasticity finite element (CPFE) simulation was carried out in this study. Thereinto, representative volume element (RVE) models of polycrystals were established according to the initial grain morphology measured by electron backscatter diffraction (EBSD). The results show that the surface roughening degree of the solid solution specimen is worse than that of annealed specimen after uniaxial tension deformation. In comparison with the annealed specimen, the grains show a larger size after solid solution treatment, thus resulting in the coarse surface to a certain extent. Moreover, texture type and density also have a significant influence on surface roughness. The rotation of grains with an S and Copper orientation intensifies the surface roughening during tensile deformation. The deformation difficulty of Goss texture in the normal direction (ND) and tangential direction (TD) varies, thus contributing to the different surface morphology. The research results will provide guidance for the improvement of the surface quality of high-strength aluminum alloy aerospace components.

## 1. Introduction

Lightweight and high-strength aluminum alloys are widely used in the aerospace manufacturing field, such as rocket fuel tanks [1,2] and aircraft extra-large panels [3,4]. Among them, 2219 aluminum alloy, with the advantages of high strength, good weldability and high fracture toughness, is a candidate material used in aerospace components [5,6]. Since surface quality is related to cracking, stress corrosion, and fatigue failure, which significantly affect the integrity and service life of aluminum alloy [7,8], it has been focused on during the precise manufacturing of aerospace components. What is more, some aluminum alloy components even fail to meet manufacturing and service requirements due to surface roughening (SR, also named surface orange peel) defects. Therefore, it is necessary to conduct in-depth research on the surface roughening behavior and mechanism of metals, thus to propose feasible solutions for predicting and controlling surface roughness in engineering applications.

In order to reveal the surface roughening mechanism after plastic forming, experimental methods such as microstructure observation are often used. Meanwhile, contact profilometry and scanning laser confocal microscopy (SLCM) are employed to measure the degree of surface roughening. In recent years, with the continuous development of crystal plasticity finite element (CPFE), many researchers have studied the causes of SR by combining their studies with a CPFE simulation [9]. It is generally believed that the internal microstructure and external forming conditions of materials are the main reasons affecting the surface roughening of aluminum alloy parts. The influencing factors of the internal microstructure on SR are grain size, the number of abnormally large grains, lattice structure, crystal orientation, and the content of internal alloying elements [10,11,12]. The external forming conditions, such as strain, strain rate, deformation gap, pre-deformation, and heterogeneous magnetic or temperature fields, also have a different influence on material surface roughening [13,14,15]. Among them, the increase in grain size on the surface or sub-surface and abnormally large grains will lead to the occurrence of a large plastic deformation difference between grains, thus leading to bad surface quality. Meanwhile, due to the difference in lattice structure and initial orientation of grains, the resistance of dislocation movement on the sliding plane of each crystal also changes with the increase in strain. The inhomogeneous deformation between grains aggravates the degree of SR. Finally, the cumulative normal displacement of grains along the thickness direction increases, resulting in surface roughening [3,16,17].

Moreover, regular surface roughening morphology features are related to the spatial distribution of crystal orientation. Korkolis et al. [18] found that surface roughening induced by plastic deformation is caused by the initial grain orientation, and the surface peaks and valleys mainly occur at the grain boundaries. This is because the grain boundaries play a role of kinematic constraint, so the extreme values of mechanical fields such as out-of-plane displacement occur at grain boundaries. Shi et al. [19] found that the banding of Cube and Goss texture components in AA6111 aluminum alloy plates were the main reasons for the formation of roping, and the roughening of roping morphology deepens with the increase in strain. If the volume fraction of the original weave component is kept constant, the surface roping roughening will be optimized with the increasing proportion of surface/near-surface grains with a random orientation.

In addition, to studying the affecting causes of surface coarsening, it is equally important to characterize the actual degree of surface coarsening of the material. The surface roughening process is coupled by multiple complex factors that jointly act on the surface of materials, and metals will present a complex surface morphology, with features such as peaks and valleys, roping, orange peel, and ring dents (produced on cylindrical rods) during plastic deformation. Although many researchers have studied the factors that affect the surface roughening of aluminum alloy parts, it is difficult to use reliable methods for quantitative description. To quantitatively characterize the degree of surface coarsening during deformation, Tran et al. [20] used the arithmetic mean surface roughness Ra and thickness uniformity parameter ξ to quantitatively evaluate the surface roughness. The results show that the formation of ridges and valleys leads to the increase of thickness inhomogeneity (ξ = 0.54). Hu et al. [21] introduced a new index to quantitatively assess the degree of roping, termed Roping Intensity (RI). Based on the 3D morphological data, this index was further utilized to evaluate four AA6XXX aluminum alloy plates exhibiting varying degrees of roping. Moreover, the RI metric can be easily adapted to other band-like features. At present, extracting the surface node coordinates of crystal plastic models and calculating the surface root mean square deviation (Sq) of the contour has become a commonly used method for calculating 3D SR.

In this paper, in order to study the surface roughening behavior and mechanism in aluminum alloy under tensile deformation, experiments and CPFE simulations are employed. First, the surface morphology of annealed and solid solution 2219 aluminum alloy after tensile deformation was compared and the roughness was quantitatively measured. Subsequently, representative volume element (RVE) models and crystal plasticity finite element models (CPFEMs) were established to explore the mechanism of surface roughening. Finally, the effects of grain size, texture, and heat treatment on surface roughening were studied. This work will provide guidance for improving the surface quality of high-strength aluminum alloy components.

## 2. Materials and Methods

### 2.1. Material

**Figure 1:**
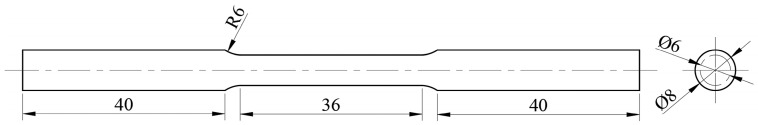
Dimensions of tensile specimen (GB/T 228.1-2021).

During the processing of aluminum alloy parts, it is found that the heat treatment state of the material has a significant influence on the surface coarsening behavior. In order to study the effect of the actual material state on the surface coarsening behavior, 2219 aluminum alloy was taken as the experimental object in this paper (its chemical composition is shown in Table 1). According to the standard GB/T 228.1-2021 [22], the bone rod-like tensile specimen as shown in Figure 1 was processed. Specimens were selected under two heat treatment states, namely an annealing sample (A-specimen) and solid solution sample (S-specimen). Therein, solid solution was applied on the A-specimens to obtain the S-specimens at 535 °C and after 1 h.

**Figure 2:**
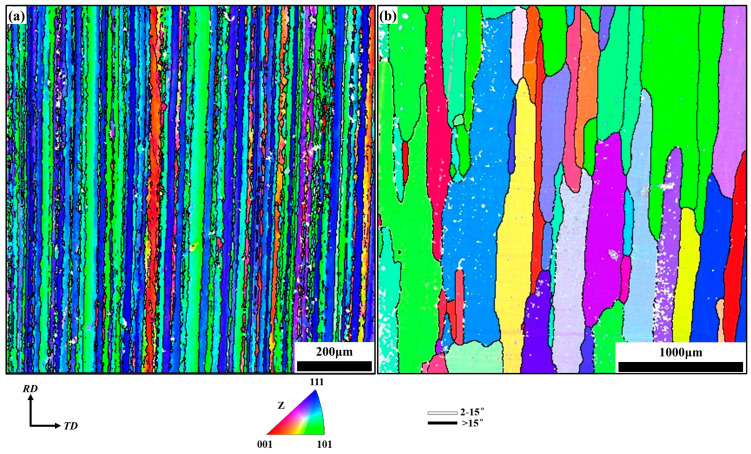
Inverse pole figure of the 2219 aluminum alloy (**a**) in the annealed state, (**b**) in the solid solution state.

Figure 2 shows the electron backscatter diffraction (EBSD) results of annealed and solid solution specimens. Different colors represent different grains. As for the A-specimen, the lath-shaped grains with a width of about 62 μm are elongated along the axial direction as shown in Figure 2a. After solid solution treatment (S-specimen), the grains grow to a large size, whose average size is about 231 μm, as shown in Figure 2b.

The typical texture volume fraction of the specimens determined by EBSD experiment is shown in Table 2, in which the Copper texture volume fraction in the A-specimen reaches 23% and the Goss and S texture in the S-specimen reach 15.2% and 18.7%, respectively. After solid solution treatment, the specimens exhibited a substantial increase in Goss and S texture, accompanied by a notable decrease in Copper texture. Additionally, there was a slight increase in Cube and Brass textures.

### 2.2. Uniaxial Tension Experiment

**Figure 3:**
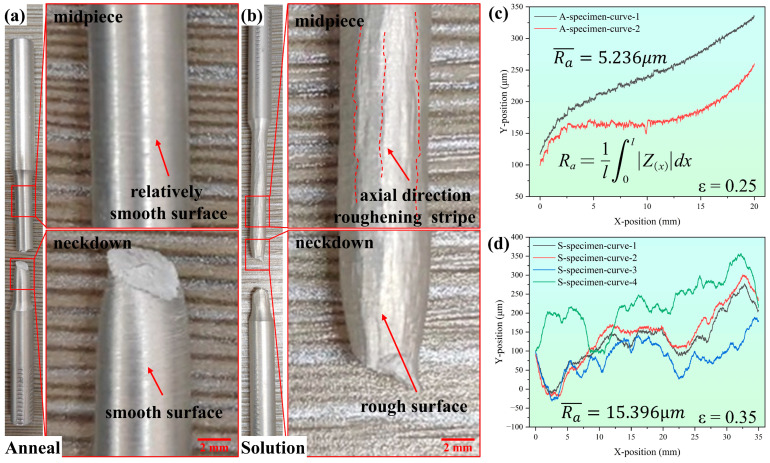
The surface morphology of 2219 aluminum alloy after tensile deformation: (**a**) the annealed specimen (A-specimen), (**b**) the solid solution specimen (S-specimen), (**c**) the A-specimen surface profile and average *R*_a_ = 5.236 μm, (**d**) the S-specimen surface profile and average *R*_a_ = 15.396 μm.

Figure 3 shows the surface morphology of 2219 aluminum alloy after tensile deformation, for which uniaxial tensile experiments were performed on a Meitesi (MST) testing machine (Meitesi, Shenzhen, China) with a tensile speed of 2 mm/min and ambient temperature of 25 °C. It can be seen that the surface of the A-specimen is relatively smooth after tensile deformation, and the specimen presents an obvious necking deformation, as shown in Figure 3a. However, the S-specimen shows a rough surface after tensile deformation. Some protruded ridge lines extend along the axial direction of the S-specimen, as shown in Figure 3b. To quantitatively characterize the surface roughness of the two specimens, we employed a contact profilometer to measure the surface roughness *R*_a_, as depicted in Figure 3c,d. The A-specimen exhibited a discernibly lower average surface roughness of 5.236 μm, while the S-specimen presented a significantly greater average surface roughness of 15.396 μm.

### 2.3. Characterization Method of Surface Roughening

In order to evaluate the influence of material states on the degree of surface roughening, three-dimensional surface topography characterization methods have often been adopted. Among them, the root mean square deviation of contour is a commonly used method to reflect the surface roughness of a three-dimensional topography [17,18]. Thus, the root mean square deviation of contour is employed in this work, whose expression is given as follows:

(1) $$S_{3q}=\sqrt{\frac{1}{MN}\sum_{i=0}^{M}\sum_{j=0}^{N}E^{2}\left(x_{i},y_{j}\right)}$$

where *M* and *N* are the number of discrete sampling points in the X-axis and Y-axis directions on the sampling area, respectively, and *E*(*x*, *y*) is the height difference between the original surface and the least squares datum surface on the sampling point. The surface displacement data of the model are extracted using a Python (version 3.12.0) script and the S_3q_ value of the model surface calculated.

## 3. Crystal Plasticity Finite Element Model

### 3.1. Crystal Plasticity Model

The precise mathematical expression of the theory of crystal deformation kinematics was given by Hill and Rice [23], and the following is a brief introduction to the theory. The plastic deformation of a crystal is generated by lattice distortion and rigid rotation and the deformation corresponding to the uniform shear of the crystal along the glide direction. The total deformation gradient **F** of the crystal can be expressed as

(2) $$F=F^{e}\cdot F^{p}$$

where $F^{e}$ represents the deformation gradient generated by lattice distortion and rigid rotation and $F^{p}$ represents the deformation gradient corresponding to the uniform shear of the crystal along the glide direction.

The velocity gradient in the current state **L** is given by a standard formula:

(3) $$L=\dot{F}F^{-1}={\dot{F}}^{e}\overset{-1}{F^{e}}+F^{e}{\dot{F}}^{p}{\dot{F}}^{p}\overset{-1}{F^{e}}=L_{e}+L_{p}$$

where $\dot{F}$ is the material derivative of **F** and $F^{-1}$ is the inverse of **F**. The velocity gradient tensor **L** can be divided into two parts, where $L_{e}$ is the velocity gradient tensor of elastic deformation and $L_{p}$ is the velocity gradient tensor of plastic deformation.

(4) $$L_{e}={\dot{F}}^{e}\cdot \overset{-1}{F^{e}}$$

(5) $$L_{p}=\sum_{\alpha =1}^{n}\overset{*}{m^{\left(\alpha \right)}}\overset{*}{n^{\left(\alpha \right)T}}\overset{\cdot }{\gamma^{\left(\alpha \right)}}$$

where $\overset{*}{m^{\left(\alpha \right)}}$ is the slip direction vector of the αth slip system in the deformed state, $\overset{*}{n^{\left(\alpha \right)}}$ is the normal direction vector of the αth slip system in the deformed state, and $\dot{\gamma^{\left(\alpha \right)}}$ is the slip shear rate of the αth slip system.

Combining the elastic theory of Hill and Rice, the plastic constitutive equation of single crystal can be obtained as

(6) $${\overset{\nabla }{\sigma }}^{e}+\sigma \left(I:D^{e}\right)=L:D^{e}$$

where **I** is the second-order identity tensor, **L** is the fourth-order instantaneous elastic modulus tensor, and ${\overset{\nabla }{\sigma }}^{e}$ represents the Jaumann derivative of the Kirchhoff stress tensor $\overset{\nabla }{\sigma }$ with the intermediate configuration as the reference state, and its component form is as follows:

(7) $${\overset{\nabla }{\sigma }}^{e}=\overset{\nabla }{\sigma }+\left(W-W^{e}\right)\cdot \sigma -\sigma \cdot \left(W-W^{e}\right)$$

where $\overset{\nabla }{\sigma }=\dot{\sigma }-W\sigma +\sigma W$.

In the case of negligible lattice elastic distortion, Schmid stress is the interfacial shear stress, which is defined as

(8) $$\tau^{\left(\alpha \right)}=m^{*\left(\alpha \right)}\cdot \sigma \cdot n^{*\left(\alpha \right)}$$

The rate of change of Schmid stress is

(9) $${\dot{\tau }}^{\left(\alpha \right)}=m^{*\left(\alpha \right)}\cdot \left[\overset{\nabla e}{\sigma }+\sigma \left(I:D^{e}\right)-D^{e}\cdot \sigma +\sigma \cdot D^{e}\right]\cdot n^{*\left(\alpha \right)}$$

Based on Schmid’s law, the slip rate ${\dot{\gamma }}^{\left(\alpha \right)}$ on the crystalline solid α slip system is determined by its corresponding decomposition shear stress $\tau^{\left(\alpha \right)}$:

(10) $${\dot{\gamma }}^{\left(\alpha \right)}={\dot{a}}^{\left(\alpha \right)}f^{\left(\alpha \right)}\left(\tau^{\left(\alpha \right)}/g^{\left(\alpha \right)}\right)$$

where the constant ${\dot{a}}^{\left(\alpha \right)}$ is the reference strain rate on the α slip system and the variable of the current strength $g^{\left(\alpha \right)}$ of the α slip system. The dimensionless function $f^{\left(\alpha \right)}$ is used to describe the stress-dependent relationship of the strain rate, expressed as follows:

(11) $$f^{\left(\alpha \right)}\left(x\right)=x{\left|x\right|}^{n-1}$$

where *x* can be regarded as $\tau^{\left(\alpha \right)}/g^{\left(\alpha \right)}$ in Equation (10) and *n* is the rate sensitivity coefficient, when the material is rate-independent.

The incremental law of strain hardening is described by the evolution of strength $g^{\left(\alpha \right)}$:

(12) $${\dot{g}}^{\left(\alpha \right)}=\sum_{\beta =1}^{n}h_{\alpha \beta }{\dot{\gamma }}^{\left(\beta \right)}$$

where $h_{\alpha \beta }$ is the instantaneous slip-hardening coefficient. When α=β, $h_{\alpha \beta }$ is called the self-hardening coefficient, also known as the self-hardening modulus; when α≠β, $h_{\alpha \beta }$ is called the potential hardening coefficient or coupling hardening coefficient, also known as the latent hardening modulus. The self-hardening modulus is

(13) $$h_{\alpha \alpha }=h\left(\gamma \right)=h_{0}\sec\;h^{2}\left|\frac{h_{0}\gamma }{\tau_{s}-\tau_{0}}\right|$$

where $h_{0}$ is the initial hardening modulus, $\tau_{0}$ is the initial critical shear stress (also known as yield shear stress), and $\tau_{s}$ is the saturated flow stress. γ is the cumulative shear strain on all activated slip systems, which can be expressed as

(14) $$\gamma =\sum_{\alpha }\int_{0}^{t}\left|{\dot{\gamma }}^{\left(\alpha \right)}\right|dt$$

The latent hardening modulus is

(15) $$h_{\alpha \beta }=qh\left(\gamma \right)\;\left(\alpha \neq \beta \right)$$

where q is a constant and is the ratio of self-hardening and latent hardening strength.

### 3.2. Determination of Constitutive Parameters

Based on the experimental results of the macroscopic tensile and EBSD data of 2219 aluminum alloy under different heat treatment conditions, the simulation model of crystal plasticity of 2219 aluminum alloy is established in this paper. The geometric size of the model is 4 mm × 1 mm × 0.1 mm, and the number of annealed grains and solid solution grains involved in the verification are 100 and 50, respectively. Random grain orientations are used in the model verification stage to reduce premature necking caused by local soft orientation aggregation. At the same time, the complete integration unit C3D8 is used in the simulation of the verification model, and the C3D8 unit is also used in the subsequent simulation, which can effectively reduce the simulation error and improve the calculation accuracy [13,24].

**Figure 4:**
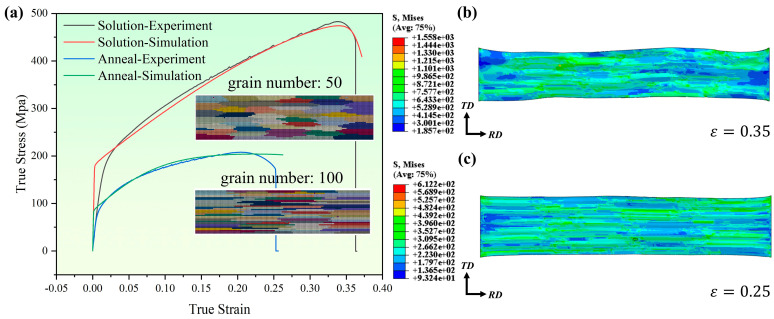
Results of (**a**) experiment and simulation comparison and von Mises stress at (**b**) ε = 0.35 in the solid solution state and (**c**) ε = 0.25 in the annealed state.

In this paper, an inverse finite element method was used to determine the crystal plasticity parameters. Namely, the constitutive model parameters were determined by fitting the experimental and simulated true stress–strain curves. Figure 4 shows the fitting of the true stress–strain curves of the polycrystalline models in the annealed state (A-specimen) and solid solution state (S-specimen). The constitutive model parameters are given in Table 3. The von Mises stress of the two models at their fracture strains is shown in Figure 4b,c.

Because an extensionometer was not used in the uniaxial tensile experiment, the slope of true stress–strain curves in the elastic phase obtained by experiment had a deviation. Thus, the plastic phase is mainly considered in the fitting of stress–strain curves. It can be found from Figure 4 that the gap between true stress–strain curves obtained based on the CPFE simulation and experiment is within 10%, which proves the reliability of the fitting parameters of the crystal plasticity model to a certain extent.

### 3.3. Establishment of Finite Element Models

The metallography and EBSD data of annealed and solid solution 2219 aluminum alloy were applied to the crystal plasticity finite element simulation. Moreover, in order to make a further study of the influencing factors of surface roughening, various grain data such as grain size, grain aspect ratio, and crystal orientation were also applied to the crystal plasticity finite element simulation [25,26]. It can be seen from Table 4 that the grain size in the annealed state is much smaller than that in the solid solution state.

**Figure 5:**
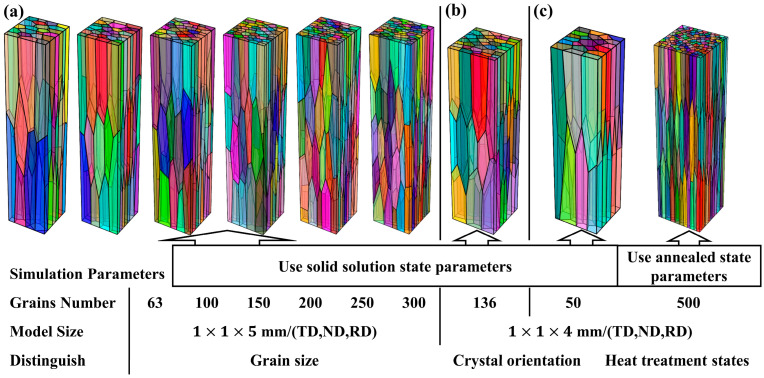
Simulation models and parameters: (**a**) to study the effect of grain size on SR; (**b**) to study the effect of crystal orientation on SR; (**c**) to study the effect of heat treatment states on SR. Different colors stand for different grains.

In addition, based on the Voronoi diagrams principle, a variety of RVE models with different grain numbers and morphological domains (Figure 5) were created with Neper [27], which is an open software for polycrystalline generation and meshing. Actually, the ratio of the number of grains contained in the annealed state and the solid solution state equal-volume RVE model is 20:1 (see Table 3 for details). Figure 5a shows the six RVE models with different grain numbers, which are used to simulate the influence of grain size on the surface roughening of the S-specimen. As the grain number increases from 63 to 300, the grain size is continuously refined. Figure 5b shows the RVE model with 136 grains, which is used to simulate the influence of different crystal orientations on the surface roughening of the S-specimen. Figure 5c shows the two RVE models under different heat treatment states, in which the left is the solid solution RVE model containing 50 grains and the right is the annealed RVE model containing 500 grains. Relevant studies have shown that when the grain number of an RVE model reaches 50, the predicted accuracy of the model is good compared with the actual conditions. If 50 grains are set in the solid solution RVE model, the annealed RVE model should contain 1000 grains; the excessive number of grains will lead to a sharp increase in the computational amount but a limited increase in accuracy. Therefore, considering the calculation simulation accuracy and efficiency, 500 grains are chosen in the annealed RVE model.

## 4. Results and Discussions

### 4.1. Effect of Grain Size on SR

In order to study the effect of grain size on the surface roughening of solid solution 2219 aluminum alloy, six cuboid RVE models with the same domain morphology and different grain sizes are established (Figure 5a). The initial size of the RVE model is 1 mm × 1 mm × 5 mm (*TD*, *ND*, *RD*), which consists of 63 grains. The grain size is similar to the actual size of solid solution grains in the experiment. Random crystal orientations are used in all six RVE models to insure the reliability of the finite element simulation.

**Figure 6:**
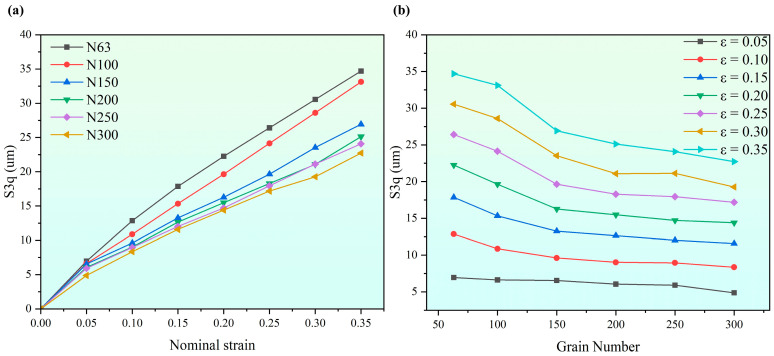
Effect of grain size and strain on surface roughening: (**a**) grain size and (**b**) nominal strain.

Equation (1) was used to calculate the S_3q_ of the simulation models under different strain and grain sizes. Figure 6 shows the effect of grain size and strain on surface roughening under uniaxial tension, in which N stands for grain number (Figure 6a) and ε stands for nominal strain (Figure 6b). As a whole, the S_3q_ decreases with the increase in grain number. Moroever, the S_3q_ does not strictly increase linearly with the increase in grain size and strain, and the growth rate of S_3q_ decreases gradually with the increase in strain. At a small tensile strain, the effect of grain size on surface roughening is not prominent. At a large tensile strain, the larger the grain size, the more severe the surface roughening. However, as the grain size continues to decrease, the decrease rate of surface roughness also gradually decreases, which means that the surface roughening phenomenon cannot be completely eliminated by grain refinement.

### 4.2. Effect of Crystal Orientation on SR

**Figure 7:**
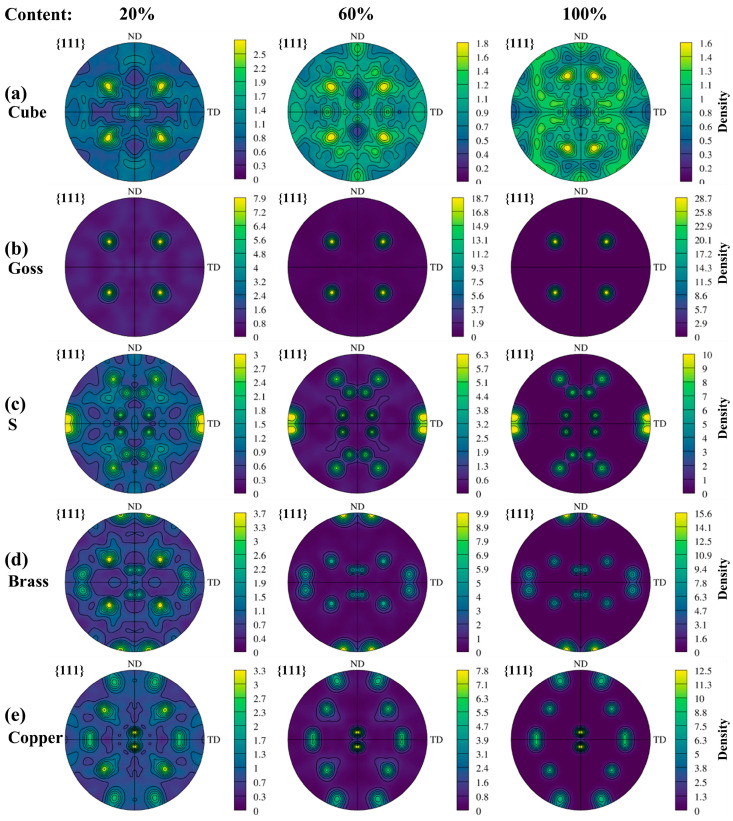
Pole figures with the content of a single typical texture increasing from 20% to 100%. (**a**) Cube texture, (**b**) Goss texture, (**c**) S texture, (**d**) Brass texture, (**e**) Copper texture.

It is well known that when the grain orientations are selective or convergent in the forming process, different textures will be generated in polycrystalline metal materials. Research studies have shown that surface roughening is also closely related to the texture of the material [28,29,30]. Since the Cube, Goss, S, Brass, and Copper textures are the typical textures produced in the initial extruded 2219 aluminum alloy, and the typical textures content is obviously changed after solid solution treatment, this work mainly studies the influence of the above textures on surface roughening. The RVE model and parameters are shown in Figure 5b and Table 3. Meanwhile, by controlling the proportion of typical texture and random orientation in the model, the stress–strain and surface roughening of the model were studied when the content of a single texture increased from 20% to 100%. Figure 7 shows {111} pole figures plotted by the initial crystal orientations used in the CPFE simulation, of which only the 20%, 60% and 100% content of a single typical texture are shown.

**Figure 8:**
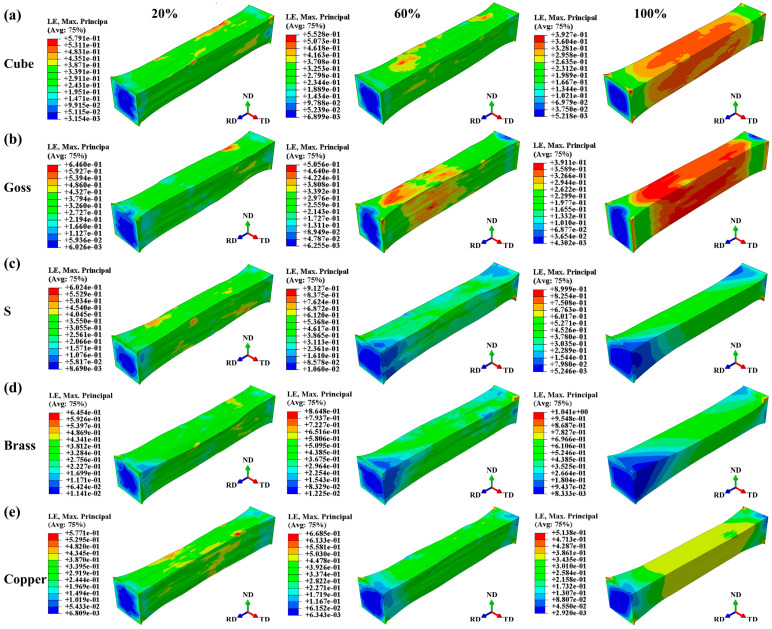
Logarithmic strain (LE) under uniaxial tension: (**a**) Cube texture, (**b**) Goss texture, (**c**) S texture, (**d**) Brass texture, (**e**) Copper texture. The texture content is 20%, 60%, and 100% in order, and ε = 0.35.

Figure 8 shows the logarithmic strain (LE) of the RVE models with different texture contents. The results show that when the texture content is low (such as a 20% texture content), most grains’ orientations are random, and the peak LE is concentrated in a small range. Under a 60% texture content, surface morphology mainly depends on the texture type. The peak LE of Goss texture is concentrated at the left end of the model, and the plastic deformation of the grain is large. This means that at the right end of the model, there are more grains with a Goss texture orientation, and the plastic deformation of grains is difficult. With the increase in the proportion of a certain crystal orientation, the distribution of LE tends to be uniform (such as a 100% texture content), and the surface morphology tends to be smooth. Models with different texture types exhibit a distinct deformation diversity in different directions. Cube texture demonstrates relatively uniform deformation in the all three directions. Goss texture shows pronounced deformation in the TD, while the deformation in the ND is less significant. S texture and Copper texture exhibit high similarity in their deformation patterns, both involving rotation around the RD. However, Brass texture displays opposite deformation patterns compared to Goss texture, with significantly lower deformation in the TD compared to RD.

**Figure 9:**
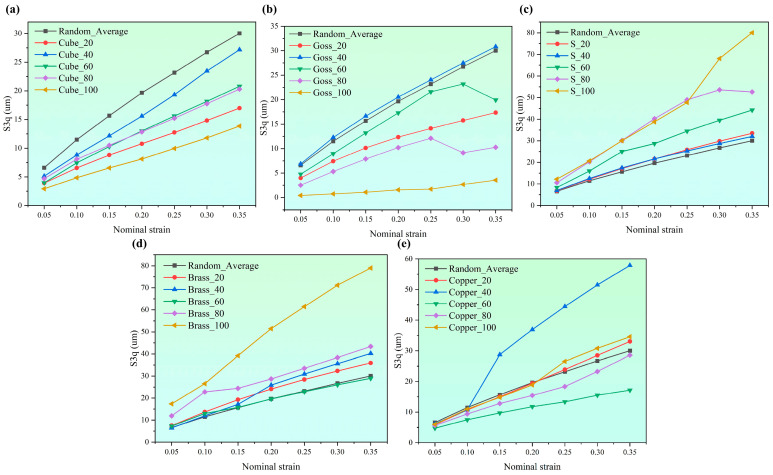
Effect of typical texture types and contents on SR: (**a**) Cube texture, (**b**) Goss texture, (**c**) S texture, (**d**) Brass texture, (**e**) Copper texture.

Figure 9 shows the influence of typical texture types and contents on SR. To compare the surface roughness with random crystal orientations, three CPFEMs with random crystal orientations were built, and the average S_3q_ of the simulation results was taken as a reference (Random_Average in Figure 9a–c). The results show that the S_3q_ values of Cube texture (Figure 9a) and Goss texture (Figure 9b) are almost lower than the average value of S_3q_ at random crystal orientations, and S_3q_ values gradually decrease with the increase in Cube and Goss texture content. On the contrary, the S_3q_ values of S texture (Figure 9c) are higher than the average value of S_3q_ at random crystal orientation, and the S_3q_ value gradually increases with the increase in S texture content. The S_3q_ values of Brass texture (Figure 9d) exceed the average S_3q_ value obtained under random crystal orientation, but they exhibit a gradual increase with an increment in the Brass texture content. For the S_3q_ values of Copper texture, see Figure 9e.

**Figure 10:**
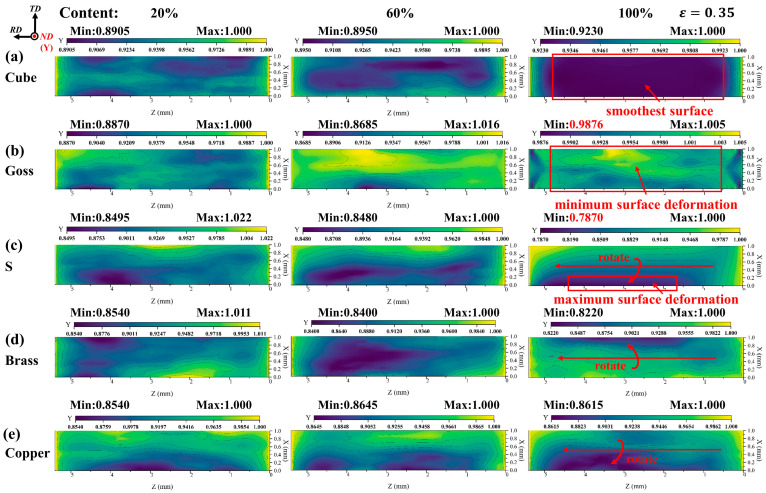
The surface morphology changes in the model with a different texture content, ε = 0.35. (**a**) Cube texture, (**b**) Goss texture, (**c**) S texture, (**d**) Brass texture, (**e**) Copper texture.

Figure 10 shows the surface morphology variation in the models with different typical texture contents, in which the color represents the coordinate components in the Y-direction. A yellow color stands for the undeformed boundary, and a blue-purple color indicates the presence of large surface displacements. The results show that the stretched surface is almost parallel to the original non-deformed surface with the increase in Cube texture content, especially when the texture content increases to 100% (Figure 10a). As the Goss texture content increases, the deformation of the stretched surface gradually decreases (Figure 10b). At the same texture content, the plastic flow in ND is more difficult for Goss texture than that for other typical textures. As the S and Copper texture content increase, it can be observed that the stretched surface rotates in the RD (Figure 10c,e). As the Brass texture content increases, the maximum deformation on the model’s surface along ND is only smaller than that of S texture (Brass texture: 0.178 mm, S texture: 0.213 mm), and the degree of surface rotation along RD is also smaller than that of S texture and Copper texture. The grain rotation can be explained by Schmid’s law. According to Schmid’s law, the orientation factor of a crystal is determined by $cos\rho_{0}cos\lambda_{0}$, where $\rho_{0}$ represents the angle between the externally applied load direction and slip plane normal and $\lambda_{0}$ represents the angle between the externally applied load direction and slip direction. It is known that $\lambda_{0}$ decreases and $\rho_{0}$ increases with increasing slip deformation. When both $\rho_{0}$ and $\lambda_{0}$ approach 45°, the crystal is in a geometrically softening state, allowing for yielding at lower loads. When $\rho_{0}$ and $\lambda_{0}$ deviate from 45°, the orientation factors are either larger or smaller than 0.5, causing crystal rotation under the externally applied loading. When the crystal rotation leads to a $\rho_{0}$ and $\lambda_{0}$ approaching 45°, the grain rotation ceases.

**Figure 11:**
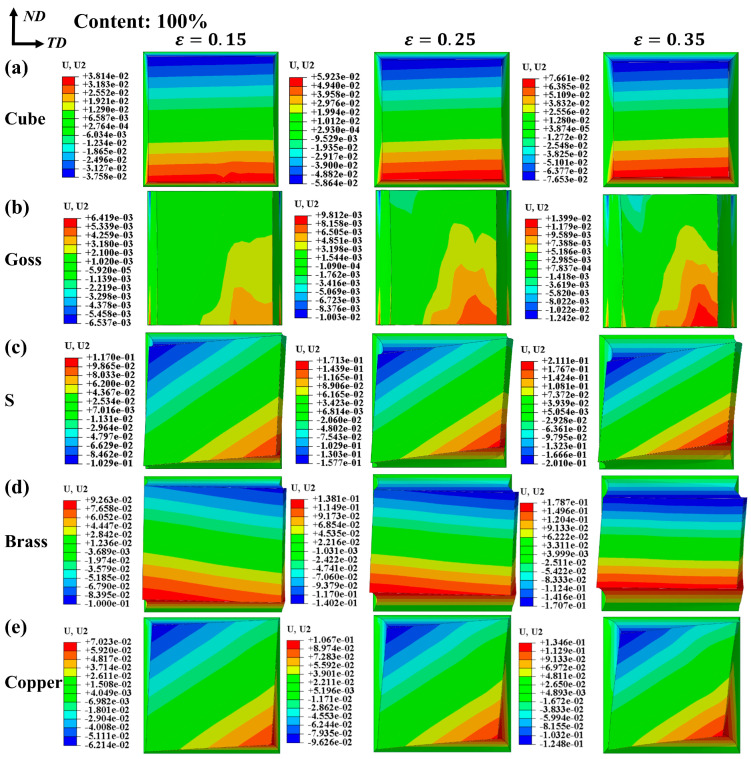
The deformation evolution of the middle section of the model under different strains shows that the texture content is 100%. (**a**) Cube texture, (**b**) Goss texture, (**c**) S texture, (**d**) Brass texture, (**e**) Copper texture.

Figure 11 shows the displacement along ND at the middle section of the model, which can reflect the deformation rule under different strains. The result shows that the model with Cube texture deforms uniformly in all directions according to the shape of the section (Figure 11a). The deformation resistance of Goss texture in the TD and ND direction is different (Figure 11b), in which the maximum deformation in ND is only 14 μm. With the increase in strain, the grains with an S orientation rotate and produce a peak region. The maximum displacement along ND is 211.1 μm (Figure 11c). The deformation direction of Brass texture is opposite to that of Goss texture. Therein, ND deformation is large, and the grains rotate slightly with the increase in strain (Figure 11d). Copper textures have peak regions similar to S textures, but the valleys formed by Copper textures are shallower, and the maximum displacement in ND is 134.6 μm (Figure 11e).

### 4.3. Effect of Heat Treatment on SR

To study the effect of heat treatment on surface roughening, CPFEMs with material parameters of a solid solution state and annealed state are used. Thereinto, the grain sizes in the RVE models are the same as the actual grain sizes measured by EBSD, as shown in Figure 5c. In order to enhance the reliability of the CPFE simulation, a subset of grains in the RVE model were assigned crystal orientations corresponding to the textures determined by EBSD result (see Table 2). Additionally, a misorientation of 5~8° was incorporated. The remaining grains were assigned random crystal orientations [3].

**Figure 12:**
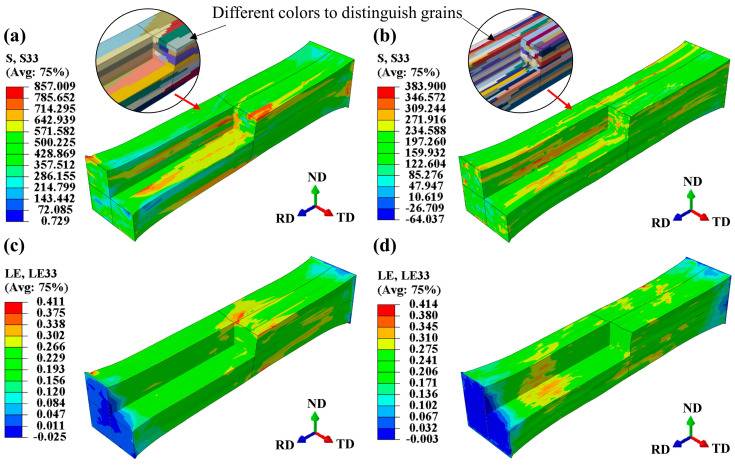
Normal stress component S33 and logarithmic strain component LE33 at ε = 0.25: (**a**,**c**) in solid solution state, (**b**,**d**) in annealed state.

**Figure 13:**
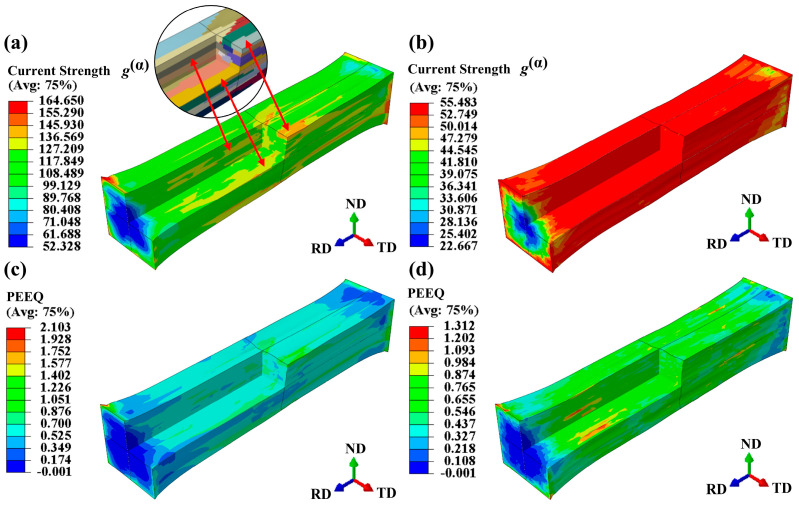
Current strength and equivalent plastic strain (PPEQ) at ε = 0.25: (**a**,**c**) in solid solution state, (**b**,**d**) in annealed state.

Figure 12 show the normal stress component S33 and logarithmic strain component LE33 under two heat treatment states in RD. Among them, the peak stress of the solid solution model reaches 857 MPa (Figure 12a), with an obvious stress concentration in some areas, while the peak stress of the annealed model is only half that of the solid solution model, reaching 384 Mpa (Figure 12b). The peak strain difference between the solid solution and annealed models is small. The grains with high strain are dispersedly distributed in the solid solution model, while the strain distribution in the annealed model is concentrated in the middle region of the model. The reason for this phenomenon is that the grain size of the annealed model is smaller, and the grain boundary density is higher in the middle of the model (the grains in the two circles in Figure 13a,b, with different colors to distinguish the grains). In the case of considering the actual crystal orientation, the texture in the model has a great influence on the stress–strain. According to Schmid’s rule, the slip deformation of soft-orientation grains is dominated by a single slip system, so the required stress is small at a certain strain, while that of hard-orientation grains is usually affected by more than two slip systems at the same time, and the required deformation is large at the same strain.

Figure 13a,b illustrate the current strength under two different heat treatment conditions. The current strength represents the resistance to dislocation motion on slip planes and is calculated through incremental updates in the UMAT subroutine. From Figure 12a and Figure 13a, the regions with a higher current strength present a higher stress in the solid solution model. However, the current strength and stress distribution in the annealed model is more uniform in Figure 12b and Figure 13b. Figure 13c,d display the equivalent plastic strain (PEEQ) under the two heat treatment conditions. A higher PEEQ value indicates greater plastic deformation in the grains. The uneven distribution of PEEQ also implies a roughening of the free surface in the RVE model. The peak PEEQ value of the solid solution model is 2.103, which is higher than that of the annealed model. In the solid solution model, PEEQ values of most of grains are below 0.525, while the grains with a higher current strength and stress values have PEEQ values above 1.051. This suggests that these grains belong to “hard orientations” and are difficult to deform. The annealed model also contains grains that are difficult to deform, but most of the grains have PEEQ values ranging from 0.001 to 0.584. Additionally, due to the smaller grain size in the annealed model, the contribution of these difficult-to-deform grains to the cumulative strain is smaller compared to the solid solution grains.

**Figure 14:**
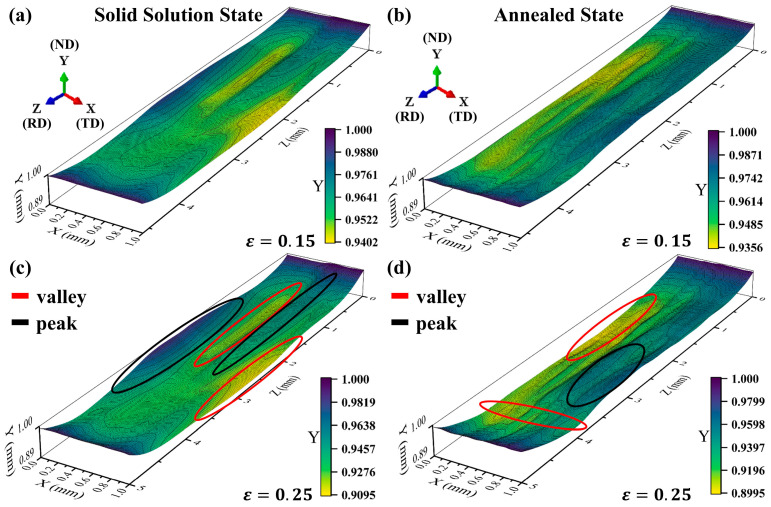
Surface morphology on top surface of solid solution model (**a**,**c**) and annealed model (**b**,**d**) at (**a**) ε = 0.15, (**b**) ε = 0.15, (**c**) ε = 0.25, (**d**) ε = 0.25. The red and black circles stand for the position of valley and peak, respectively.

By extracting coordinates on the top surface of the RVE model, a three-dimensional surface morphology of the two models was obtained under different nominal tensile strains (Figure 14). The results show that the surface roughening degree turns out to be severe with the increase in nominal strain. There are obvious peak and valley characteristics on the surface of the solid solution model (Figure 14a,c). Due to the finer annealed grains, the surface of the annealed model is more smooth under the same nominal strain (Figure 14b,d).

**Figure 15:**
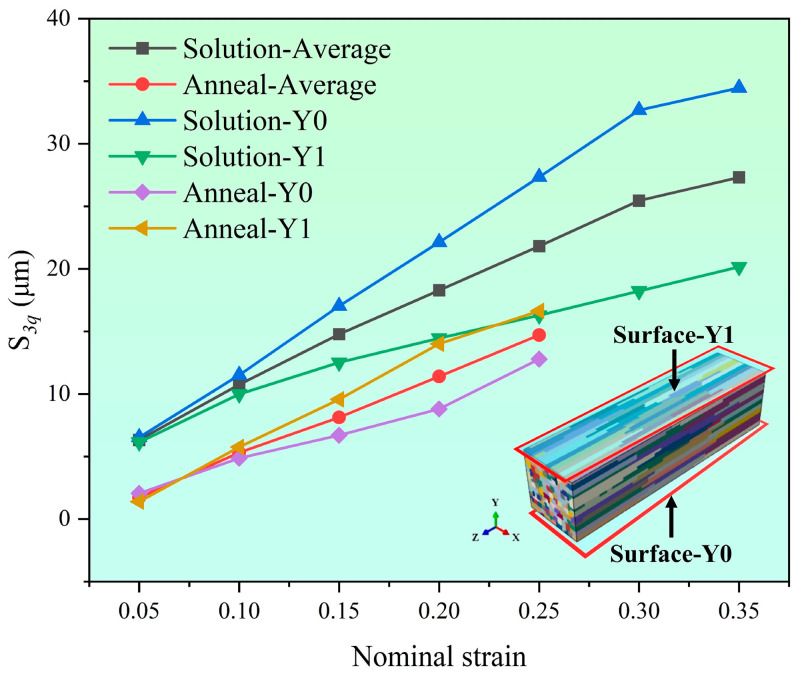
S_3q_ values of different material states on surface roughening.

The S_3q_ value of the model’s contour under different deformation degrees and different heat treatment states can be calculated by Equation (1). The S_3q_ value increases with the increase in nominal strain as shown in Figure 15, in which the S_3q_ value of the solid solution model is always larger than that of the annealed model. Due to the reduction of grain number from 1000 to 500 in the annealed model, the calculated S_3q_ value is too large, but its deviation is small.

**Figure 16:**
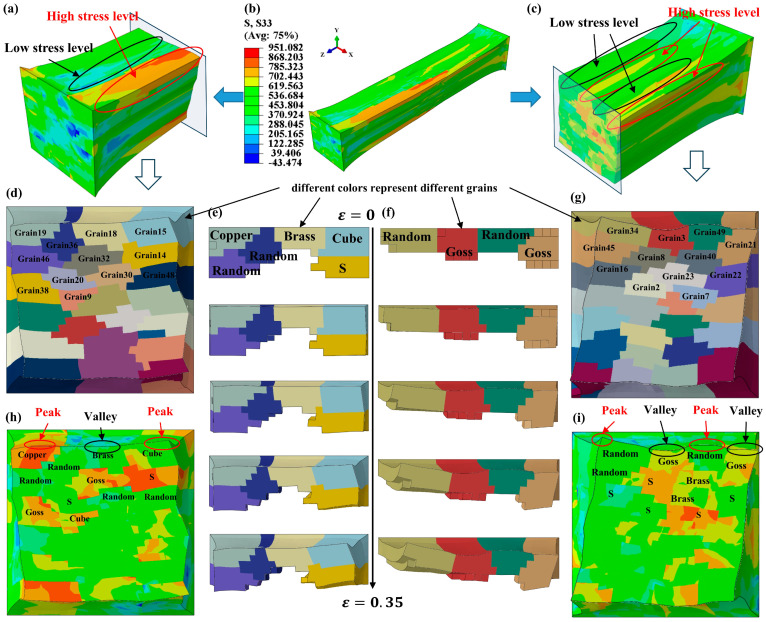
Surface roughening mechanism of solid solution model: (**a**) the left RVE model, (**b**) the normal stress component S33 at ε = 0.35, (**c**) the right RVE model, (**d**,**g**) the grains in cross-section, (**e**,**f**) five typical orientations on the near-surface, (**h**,**i**) grain orientation distribution on the near-surface.

Compared with the top-surface morphology of the solid solution model as shown in Figure 14, the surface coarsening mechanism of the solid solution model was analyzed in detail, as shown in Figure 16. We extracted the grain index and corresponding initial crystal orientations on the ND×TD cross-section (Figure 16d,f–h), which exhibited a significant stress concentration and pronounced surface roughening, as depicted in Figure 16a,b. The results shows that roughening defects of peaks and valleys occur on the top surface due to the existence of five typical orientations (Figure 16e,f). With the increase in strain, the grains with Copper and Brass orientation rotate slightly around RD, and the grains with a Cube orientation are rotated by the S orientation grains below, resulting in the top surface roughening (Figure 16e). This indicates that the orientation of near-surface grains also has a certain influence on the coarsening of the free surface. Moreover, Figure 16f shows that the grains with random orientations on the left side undergo a large rotation, while the grains with a Goss orientation have no obvious rotation around RD. Due to the constraint effect of the grain boundary, the grains with a Goss orientation have a large displacement in both ND and TD; the displacement of grains in TD is significantly larger than in ND, which in turn verifies the results of Figure 8b and Figure 11b. Roughening defects of peaks and valleys exist on the top surface of the model due to the existence of five typical textures (Figure 16h,i). Because the volume fraction of Goss and S textures are the largest of the five typical textures, there are a large stress concentration at the aggregation of Goss and S textures (Figure 16i); most of the grains have obvious rotation. The volume fractions of Cube, Brass, and Copper textures are small and make a small contribution to the cumulative strain compared to Goss and S textures.

**Figure 17:**
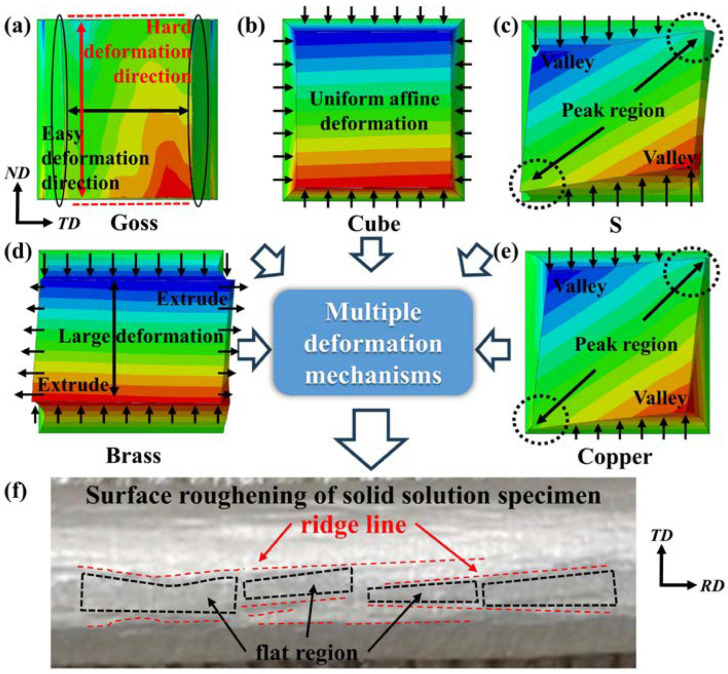
Multiple deformation mechanism-related SR under the effect of five typical textures: (**a**) Goss texture, (**b**) Cube texture, (**c**) S texture, (**d**) Brass texture, (**e**) Copper texture, (**f**) the surface roughening of solid solution specimen.

Owning to the different slip abilities of each crystal orientation, the deformation mechanisms of a material with diverse textures are different during uniaxial tension. In Figure 17a, the deformation resistance of Goss texture in the TD and ND directions is different, easy to deform in TD and hard to deform in ND. In Figure 17b, Cube texture presents an even deformation in all directions. However, S texture produces peak regions during deformation as shown in Figure 17c. In Figure 17d, the easy deformation direction of Brass texture is opposite to that of Goss texture, and large deformations occur in ND. In Figure 17e, Copper textures have peak regions similar to S textures, but the valleys formed by Copper textures are shallower. In the texturization-enhanced material, the deformation heterogeneity between grains is aggravated. Thus, surface roughening defects (such as ridge line, etc.) easily occur. As shown in Figure 17f, the surface ridge lines are formed at the grain boundary between two grains, while the flat region enclosed by the ridges consists of a single grain.

## 5. Summary and Prospect

In the manufacturing of aluminum alloy parts, it was found that the surface quality of annealed and solid solution 2219 aluminum alloy was different, and the solid solution 2219 aluminum alloy showed an obvious surface roughening phenomenon. In order to understand the causes of surface roughening under tensile deformation, a crystal plasticity finite element (CPFE) simulation along with experiments was conducted in this paper. The following can be concluded:

(1) After tensile deformation, the annealed specimen presents a comparatively low surface roughness, as indicated by an Ra value of 5.236 μm. Conversely, the solid-solutioned specimen presents a rougher surface, with an Ra value of 15.396 μm. The growth of lath-shaped grain after solid solution treatment plays a significant role in the severely roughened surface.
(2) The surface roughening degree increases with grain size and tensile strain. At a small tensile strain, the impact of grain size on surface roughening is not prominent. However, at a large tensile strain, the larger the grain size, the more severe the surface roughening.
(3) Textures also have a great influence on surface roughness, related to their diverse deformation mechanisms. The rotation of grains with an S and Copper orientation intensifies the surface roughening during tensile deformation. The deformation difficulty of Goss texture in the normal direction (ND) and rolling direction (TD) varies, leading to valley roughening at the free surface with Goss orientation grains. With the enhancement of texture, the deformation nonuniformity effect between the free-surface and near-surface grains is intensified, leading to the coarsening of free surfaces.
(4) In future studies, a more meticulous CPFEM, considering the role of second-phase particles and gradient microstructure, can be established to study their impact on surface roughness. Morever, the simulation and experiment can be carried out at actual complex plastic deformation condition such as a high strain rate.

## Figures and Tables

**Table 1:** Chemical composition of 2219 aluminum alloy (Wt/%).

Si	Fe	Cu	Mn	Mg	Zn	Ti	Zr	V	Other	Al
0.03	0.07	6.19	0.28	0.01	0.01	0.04	0.12	0.10	0.15	Bal.

**Table 2:** Typical texture volume fraction of specimens measured by EBSD experiment.

Texture Type	Euler Angle (°)			Texture Volume Fraction (%)	
	$\varphi_{1}$	Φ	$\varphi_{2}$	A-Specimen	S-Specimen
Cube	0	0	0	6.25	8.02
Goss	0	45	90	3.92	15.20
S	59	37	63	3.90	18.70
Brass	30	45	90	8.29	9.55
Copper	90	35	45	23.0	8.89

**Table 3:** Crystal plasticity material parameters of 2219 aluminum alloy.

Material Property	Symbol	Anneal State Value	Solution State Value	Unit
Elasticity constant	C11	97	97	GPa
	C12	41.5	41.5	GPa
	C44	27.7	27.7	GPa
Rate sensitivity coefficient	n	15	15	-
Reference shear strain rate	$\dot{\gamma_{0}}$	0.001	0.001	$s^{-1}$
Initial hardening modulus	$h_{0}$	135	136	MPa
Saturation flow stress	$\tau_{s}$	54	164	MPa
Initial critical shear stress	$\tau_{0}$	22.8	52.5	MPa

**Table 4:** Partial statistical data of grains under different heat treatment states.

Type	Annealed Grain	Solid Solution Grain	Unit
Average grain size	40×40×980	200×100×1600	μm/(TD,ND,RD)
Average aspect ratio of grain	(1:1:25)	(2:1:16)	-
Grain volume ratio	20:1		-

## Data Availability

The original contributions presented in this study are included in the article. Further inquiries can be directed to the corresponding authors.
